# Unraveling the Impact of COVID-19 Lockdowns on Youth Sports and Physical Activity: Insights From a Retrospective Cohort Study in Italy

**DOI:** 10.1155/ijpe/1339516

**Published:** 2025-11-06

**Authors:** E. Lodi, P. A. Gasparini, A. Brusini, M. L. Poli, G. Savino, M. G. Modena

**Affiliations:** ^1^P.A.S.C.I.A. Center (Heart Failure, Pediatric, and At-Risk Heart Disease Care Program), University Hospital Policlinico of Modena, Modena, Italy; ^2^CHIMOMO Department, University of Modena and Reggio Emilia, Modena, Italy; ^3^Sports Medicine Service, Modena Local Health Authority (Azienda USL di Modena), Modena, Italy

## Abstract

**Background:**

The COVID-19 pandemic led to widespread societal disruptions, including lockdowns aimed at reducing virus transmission. These measures had a profound impact on physical and mental health, particularly among children and adolescents. The closure of sports facilities and restrictions on outdoor activities resulted in a significant decrease in physical activity levels among this age group.

**Methods:**

A cross-sectional survey was conducted among 211 participants attending sports medicine clinics in Northern Italy. The survey is aimed at assessing physical activity levels during three distinct lockdown periods. It gathered data on demographics and tracked trends and changes in physical activity throughout these phases.

**Results:**

During the initial lockdown, 37.4% of the youth maintained regular physical activity, primarily at home, with an average of 4 h/week. By October 26, 69.6% of the participants were still engaged in physical activities, with participation stabilizing at 71.0% by the time of the survey. Inferential analyses confirmed that the increase in participation between the first and second phases was statistically significant (*p* < 0.001, Cohen's *h* = 0.85), whereas later changes were not significant. Although there was a slight increase in the weekly hours of physical activity, these levels remained below the World Health Organization's recommended guidelines. Average weekly hours of activity showed only a small, nonsignificant increase (*p* = 0.12, Cohen's *d* = 0.20). Importantly, in all phases, fewer than 20% of participants met WHO recommendations.

**Conclusions:**

The COVID-19 lockdowns significantly disrupted youth physical activity, which was heavily reliant on organized sports clubs. Despite some recovery after the restrictions were lifted, physical activity levels often fell below recommended guidelines. The disruption in physical activity patterns during the pandemic could have long-lasting effects on future health outcomes. The habits established during childhood and adolescence often persist into adulthood, and the significant drop in physical activity during the pandemic may impact health for decades. This highlights the need for targeted strategies and enhanced physical education programs to mitigate the health risks associated with sedentary behaviors.

## 1. Introduction

The COVID-19 pandemic led to significant societal disruptions, including the implementation of lockdowns to curb the virus spread. From a clinical point of view, the overall COVID-19-related mortality was significant [1, 2], with more than 6.5 million confirmed deaths and approximately 700 million patients infected by SARS-CoV-2 [3], including those requiring intensive care [4], even among the pediatric population [5]. Moreover, there has been an enormous burden on hospitals, not only due to acute admissions but also for screening purposes [6], return-to-play evaluations [7, 8], and long COVID-19–related follow-up [9, 10].

While effective in reducing transmission rates, these lockdowns had adverse effects on the physical and mental well-being of the population, especially among children and adolescents. A surge in cases of lack of confidence, anxiety, and depression was observed among clinicians and the general population [11, 12]. Furthermore, social and economic inequalities became more pronounced, particularly among workers who could not maintain their livelihoods without exposing themselves and their clients to infectious risks [13].

Sports activities were among the social and economic activities most affected by the restrictions, as sports participation faced unprecedented disruption due to facility closures and suspension of competitions. The closure of sports facilities and limitations on outdoor activities severely reduced opportunities for regular physical activity, which is crucial for maintaining cardiovascular health and psychological well-being [14, 15]. As a result, children and athletes had to adjust their lifestyles.

This article examines the effects of COVID-19 lockdowns on the physical activity patterns of children and adolescents, the transition to alternative forms of exercise, and the broader implications for maintaining youth athletic performance and health during prolonged periods of restricted movement.

This study is aimed at examining how physical activity levels among children and adolescents changed during three distinct phases of Italy's COVID-19 lockdowns. In addition to tracking participation in organized sports, the study investigated overall trends in physical activity, including shifts to individual, home-based, or digital forms of exercise. The research further sought to identify whether participation levels returned to prepandemic norms and to explore the implications of these patterns for long-term youth health.

## 2. Methods

A cross-sectional survey was conducted among 211 children and adolescents attending sports medicine clinics in Northern Italy between March 9 and October 26 of 2020. All participants were undergoing preparticipation screening for competitive sports and were aged between 6 and 18 years.

The data collection instrument was a structured questionnaire developed by the research team specifically for this study. The questionnaire was informed by existing literature on youth physical activity during the COVID-19 pandemic and was pilot-tested on a small group of 15 participants to ensure clarity and relevance. Although not previously validated, the questionnaire demonstrated good internal consistency (Cronbach's alpha = 0.81) for items related to physical activity frequency and duration.

The survey included the following domains:

• Demographics (age, sex, and previous sports experience)
• Type of physical activity engaged in during three distinct lockdown periods
• Hours per week of physical activity
• Participation in organized sports versus individual or home-based activities
• Use of online or digital exercise programs

The three study periods corresponded to major phases of the national lockdown:

• First lockdown (March 9–May 5, 2020): suspension of most social and economic activities, including sports [16].
• Second period (May 6–17, 2020): limited reopening for individual outdoor exercise (e.g., walking and running) [17].
• Third period (May 18–October 26, 2020): partial reopening of sports clubs for noncontact individual sports before reclosure of team-based activities [18].

The hours dedicated to physical activity during school hours were not considered, as there were inconsistencies even before the COVID-19 pandemic, and during the lockdown periods, many classes were unable to conduct physical education sessions either in the gymnasium or outdoors [19]. Descriptive statistics were used to identify trends in physical activity across lockdown phases. In addition to descriptive statistics, inferential analyses were performed. Proportions of participants engaging in regular physical activity and those meeting WHO guidelines across the different lockdown phases were compared using two-proportion *z*-tests. Effect sizes were expressed as Cohen's *h*, and 95% confidence intervals (95% CIs) were calculated using the Wilson method. For continuous variables (hours of physical activity per week), differences across timepoints were assessed with repeated-measures tests (paired *t*-tests or nonparametric equivalents as appropriate), and effect sizes were calculated (Cohen's *d*). A significance level of *p* < 0.05 was adopted.

### 2.1. Ethical Considerations

This study was conducted in accordance with the ethical standards outlined in the Declaration of Helsinki and relevant national guidelines for research involving minors. Informed consent was obtained from parents or legal guardians of all participants, and assent was secured from the children and adolescents themselves. Participation was entirely voluntary, and all data were collected and analyzed anonymously to protect confidentiality and minimize potential risk.

## 3. Results

Demographics and participant characteristics were as follows:

• Participants: 211 individuals
• Mean age: 12.81 years (range: 6–18 years)
• Gender distribution: 44.54% male

**Figure 1:**
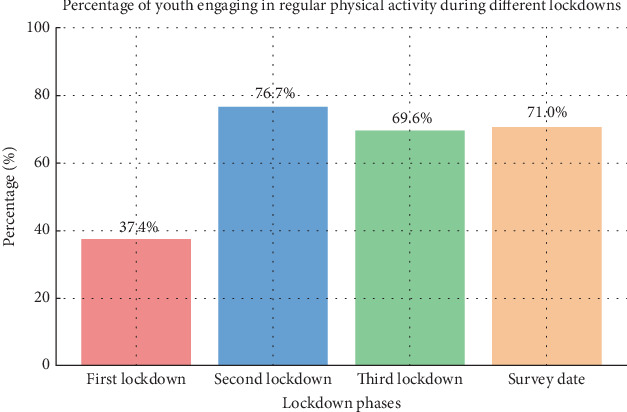
The impact of COVID-19 lockdowns on physical activity among the youth participants in the study during different phases of the COVID-19 lockdowns.

**Figure 2:**
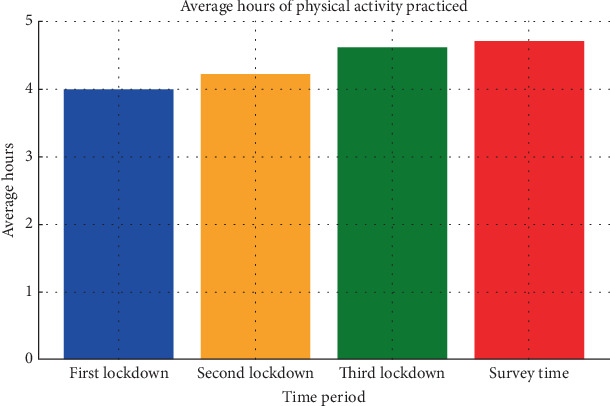
Average hours per week of physical activity practiced during different lockdown periods and at the survey time.

Impact of COVID-19 lockdowns on physical activity (Figures 1 and 2) is as follows:

• Initial lockdown (first study period):
  ◦ Physical activity participation: 37.4% of youth engaged in regular physical activity.
  ◦ Type of activity: primarily physical activity at home.
  ◦ Average hours: 4 h/week.
• Postlockdown resumption:
  ◦ Percentage engaging in physical activity: increased to 76.7%.
  ◦ Activities at sports clubs: 60.1% of those active participated in sports clubs.
  ◦ Average hours: 4.2 h/week.
• Later periods:
  ◦ October 26: 69.6% still engaged in physical activities.
  ◦ Average hours (October 26): 4.6 h/week.
  ◦ Survey date: 71.0% were engaged in physical activity.
  ◦ Average hours (survey date): 4.7 h/week.

Sports club participation was as follows:

• Percentage engaging in sports clubs: 78.3% postlockdown.
• Current engagement: 65.8% engaged in activities within a sports club.
• Continued participation: 92.6% of those active after October 26 were involved in sports clubs.

Types of sports were as follows:

• Popular sports: Volleyball (21.3%) and soccer (19.4%) were the most common.

Physical activity levels were as follows:

• Hours of physical activity: gradually increased over study periods: 4.0, 4.2, 4.6, and 4.7 h/week
• Meeting WHO guidelines: generally below recommended levels

Overall engagement statistics were as follows:

◦ Nonparticipation: 38.1% of Italians do not engage in any sports activities.
◦ Age group differences: Engagement levels varied across age groups, generally decreasing during lockdowns.

This study examines the impact of COVID-19 lockdowns on physical activity among 211 participants with a mean age of 12.81 years in Italy. The cohort comprised 44.54% males, with an average of 4.7 years of prior participation in competitive sports.

Data analysis revealed that during the initial lockdown, only 37.4% of youth engaged in regular physical activity, primarily at home due to restrictions on outdoor activities and the closure of sports clubs. Following the easing of restrictions, participation rose to 76.7%, with 78.3% of active participants (60.1% of the total) resuming activities within sports clubs. This percentage stabilized around 69.6% by October 26, increasing slightly to 71.0% at the survey date, with 65.8% engaging in sports club activities (Figure 1).

During the first lockdown, participation dropped sharply: Only 37.4% of respondents reported engaging in regular activity (95% CI: 31.0%–44.2%), with most exercises carried out at home and averaging just 4.0 h/week. Once restrictions were lifted, activity levels rebounded markedly. Participation rose to 76.7% (95% CI: 70.4%–82.0%), a change that was highly significant (*p* < 0.001, Cohen's *h* = 0.85). A large share of this recovery was driven by the reopening of sports clubs, with 78.3% of active participants (60.1% of the total sample) resuming structured activities.

In the following months, levels of engagement remained relatively stable. By late October, 69.6% of participants were still active (95% CI: 63.0%–75.6%), and at the final survey, 71.0% (95% CI: 64.4%–76.8%) continued to report regular activity. These differences, however, were not statistically significant compared to the postlockdown rebound (*p* = 0.74, Cohen's *h* = 0.03).

With respect to WHO guidelines (≥60 min/day), the proportion of participants meeting recommendations remained consistently low: 18.9% (95% CI: 14.1%–25.0%) in the first lockdown, 15.4% (95% CI: 11.0%–21.1%) after reopening, 14.9% (95% CI: 10.6%–20.6%) by October 26, and 16.6% (95% CI: 12.1%–22.3%) at the survey. No statistically significant differences were observed across these timepoints (*p* > 0.05, Cohen's *h* < 0.10). Average weekly hours of physical activity showed a modest increase from 4.0 h/week during the initial lockdown to 4.7 h/week at the time of the survey. However, this change did not reach statistical significance (*p* = 0.12, Cohen's *d* = 0.20). Importantly, despite these improvements and even at their peak, compliance with WHO recommendations (≥60 min of physical activity per day) remained consistently low. Only 18.9% of participants met the target during the lockdown, and proportions declined slightly in later phases: 15.4% after reopening, 14.9% by October, and 16.6% at the survey. No significant differences were observed across time points (all *p* > 0.05, Cohen's *h* < 0.10).

Overall, the findings reveal a striking pattern: While the reopening of sports clubs restored much of the lost participation, total activity levels never approached recommended thresholds. Even at their peak, participants averaged around 40 min/day—well below the WHO standard of 60 min. This underscores the lasting impact of the pandemic on youth physical activity behaviors, highlighting the vulnerability of this age group to structural barriers and the importance of targeted interventions to sustain adequate activity levels.

### 3.1. Types of Sports and Timing of Participation

Before the onset of the pandemic, volleyball (21.3%) and soccer (19.4%) were the most commonly practiced sports among participants. However, during the strict lockdown period (March–May 2020), participation in organized team sports ceased entirely due to government mandates. Activities during this time were primarily home-based or outdoor individual exercises (e.g., walking, jogging, and stretching). As restrictions eased in late May and early summer, sports clubs reopened for individual and noncontact training. Resumption of volleyball and soccer activities occurred primarily in the third study period (May 18–October 26), under modified guidelines such as outdoor-only practices, small group sessions, and physical distancing. This resurgence explains the continued popularity of these sports in the postlockdown data.

In Italy, participation in sports activities among young people is primarily associated with involvement in sports clubs, indicating a notable shift away from independent physical activity.

The average weekly hours of physical activity ranged from 4.0 to 4.7 across study periods. Specifically, the average hours of physical activity practiced were 4.0 during the first study period, 4.2 h in the second, 4.6 h in the third, and 4.7 h at the time of the survey (Figure 2). Analysis of standard deviations shows that, in general, activity levels were below the recommended average according to the latest WHO guideline of 60 min of moderate to vigorous physical activity per day across study periods. The percentage of youths who reached the minimum average of 60 min of moderate to vigorous physical activity per day was 18.9% in the first study period, 15.4% in the second, 14.9% in the third, and 16.6% at the time of the questionnaire.

The CONI (Italian National Olympic Committee) [20] indicated that 38.1% of people in Italy do not engage in any sports activities, with the percentage being lower in younger age groups. For example, in the 3–5 age group, 28.2% of males and 21.0% of females consistently engaged in sports, increasing in the 6–10 age group (62.2% males and 58.7% females), 11–14 (64.5% males and 56.8% females), 15–17 (58.3% males and 44.6% females), and 18–19 (50.7% males and 33.2% females). These percentages significantly decreased during the lockdown, considering that the participants usually practiced sports on average for 4.7 years prior to the study. Furthermore, the global pandemic has led to a notable decline in physical education in academic institutions, thereby exacerbating the challenges associated with promoting optimal motor development among young individuals. Prior research has demonstrated that physical education, in its current form, is an ineffective means of stimulating motor development in young people.

The data highlight significant fluctuations in youth physical activity levels during and after COVID-19 lockdowns, emphasizing the critical role of sports clubs in maintaining activity levels. It is of paramount importance to implement strategies that can mitigate the disruptions to physical activity, particularly during periods of restricted mobility, in order to promote healthy lifestyles among the younger generation.

## 4. Discussion

The COVID-19 pandemic profoundly affected physical activity levels among children and adolescents, primarily as a consequence of the suspension of organized sports and restrictions on outdoor activities. Our findings show that the most pronounced decline occurred during the initial lockdown, when participation in regular physical activity dropped to less than 40%. Following the easing of restrictions, engagement nearly doubled, representing a statistically significant improvement with a large effect size. However, once restrictions were partially lifted, subsequent fluctuations in activity levels were small, not statistically significant, and of negligible effect size, suggesting a stabilization rather than a true recovery. Despite this partial rebound, absolute levels of physical activity remained consistently below international health recommendations. Across all phases of the study, fewer than one in five participants met the World Health Organization guideline of at least 60 min of daily activity, and this proportion did not improve over time. Similarly, the modest increase in weekly training volume, from 4.0 to 4.7 h/week, did not reach statistical significance and corresponded only to a small effect size. These results indicate that, while statistical differences were detectable between phases, they did not translate into clinically meaningful improvements, as the overall levels of activity remained insufficient to meet recommended targets.

The persistence of inadequate physical activity levels highlights the vulnerability of a system heavily reliant on organized sports clubs. The temporary closure of such institutions during lockdowns left many young people without viable alternatives, and home-based or independent activities only partially compensated for this deficit. This dependency raises concerns about resilience in the face of future disruptions, whether due to public health crises or other societal challenges.

Beyond the quantitative dimension, the reduction in physical activity during the pandemic carries important implications for the broader well-being of young individuals. Physical exercise in childhood and adolescence is not only essential for maintaining fitness and cardiovascular health but also plays a central role in supporting cognitive, emotional, and social development. The interruption of structured activities therefore risks long-term consequences, including the consolidation of sedentary habits and missed opportunities for psychosocial growth.

These findings underscore the need for public health strategies that extend beyond the reinstatement of organized sports. While restoring access to sports clubs remains essential, equal emphasis should be placed on fostering independent, sustainable forms of physical activity that can be maintained regardless of external restrictions. Such interventions could help reduce reliance on institutional frameworks and strengthen the capacity of children and adolescents to achieve and sustain adequate activity levels, thereby closing the persistent gap with international health guidelines.

### 4.1. Impact on Physical Activity Levels and Sports Participation

From a physical health perspective, the benefits of regular exercise during youth and adolescence are well documented. It supports the development of strong bones and muscles, improves cardiovascular fitness, and helps maintain a healthy weight. Additionally, physical activity has been shown to alleviate symptoms of anxiety and depression, thereby improving overall mental health. These benefits extend beyond adolescence, significantly reducing the risk of chronic diseases such as cardiovascular conditions, diabetes, and obesity in later life.

Physical activity plays a crucial role in the development of young people, extending far beyond just physical health. Engaging in sports and other forms of exercise helps foster essential social skills such as teamwork, cooperation, communication, and leadership. These activities also offer young individuals opportunities to build friendships, strengthen community ties, and enhance their sense of social inclusion. Furthermore, regular physical activity encourages peer relationships, promotes inclusivity, and nurtures a sense of belonging, all of which are vital for adolescents' social and emotional well-being.

However, the COVID-19 pandemic disrupted these positive trajectories. The lockdowns and restrictions led to the abrupt cessation of organized sports, the closure of recreational facilities, and limitations on outdoor activities. As a result, physical activity levels among young people saw a significant decline. During the first lockdown, only 37.4% of children and adolescents engaged in regular physical activity, primarily within their homes. Although physical activity levels partially recovered with the easing of restrictions and the reopening of sports clubs, they remained below prepandemic norms and the WHO's recommendations.

This decline in physical activity was not isolated to one region; studies from countries like Spain, the United Kingdom, and the United States reported similar trends. For example, research by López-Bueno et al. [21] indicated that only 20% of children in Spain met the WHO's recommended activity levels during the lockdown, down from 40% prepandemic. Similarly, a survey in the United Kingdom found that only 19% of children were achieving the recommended 60 min of physical activity per day during the lockdown, compared to 47% before the pandemic [22]. These findings highlight a widespread decline in youth physical activity during the pandemic.

Several factors contributed to this decline. The closure of schools and sports clubs removed key opportunities for structured physical activity, such as physical education classes and organized sports. Restrictions on outdoor activities limited chances for unstructured play, which is also important for physical activity. The shift to remote learning and increased screen time further promoted sedentary behaviors, reducing opportunities for physical exercise [23].

The disruption in physical activity patterns during the pandemic could have long-lasting effects on future health outcomes. The habits established during childhood and adolescence often persist into adulthood, and the significant drop in physical activity during the pandemic may impact health for decades. The failure to meet the WHO's physical activity guidelines during this critical period raises concerns about a generation that may be less physically active and more prone to chronic health conditions.

The pandemic also had a profound impact on youth sports participation, a key component of physical activity for many. The cancelation of sports seasons and the closure of sports clubs led to a 50% drop in youth sports participation, particularly affecting team sports [24].

The loss of sports participation resulted in several negative outcomes. Physically, the absence of regular training and competition led to declines in fitness, including cardiovascular endurance and muscle strength [25]. Mentally, the loss of sports as an outlet for stress relief and social interaction contributed to increased anxiety, depression, and feelings of isolation among young athletes [26–29]. The disruption also affected routines and goals, creating uncertainty about the future of many young athletes.

The decline in sports participation also has wider public health implications. Regular participation in sports has been linked to a reduced risk of developing chronic diseases such as obesity, diabetes, and cardiovascular conditions. Consequently, the reduction in youth sports during the pandemic may have long-term health consequences for this generation [30].

### 4.2. Dependence on Sports Clubs and the Effect of the Lockdown

One of the most significant findings of the study is the heavy reliance of Italian youth on organized sports facilities like clubs and gyms. Among those who resumed physical activity after the lockdown, 78.3% did so mainly through these sports clubs. This reveals a critical dependence on structured environments for maintaining physical activity, as shown by the substantial drop in activity levels when these facilities were closed. To address this, it is essential to promote independent physical activities that do not rely on organized settings. This would help ensure that young people can stay active even during emergencies.

The situation in Italy is part of a larger global trend. Similar declines in youth physical activity have been observed in other countries, including Spain and the United Kingdom, due to pandemic-related restrictions. This indicates that the challenges in maintaining physical activity during the pandemic are widespread.

However, recovery patterns varied by region. In areas where outdoor activities were promoted as safe alternatives during the pandemic, a more significant rebound in physical activity was observed. This highlights the role of effective public health messaging and the availability of outdoor spaces in influencing youth activity levels.

The dependence on organized sports facilities in Italy has exposed vulnerabilities in the youth sports system, which needs to be more flexible and resilient. Additionally, the pandemic has underscored the potential of technology to support physical activity when traditional options are unavailable [31]. National consensus documents have highlighted the potential of telemedicine and remote interventions to maintain physical activity when in-person participation is not possible, especially in pediatric and adolescent populations [32, 33]. Fitness apps, virtual exercise programs, and online sports classes have become valuable tools. Nonetheless, more research is needed to determine their effectiveness in sustaining youth physical activity.

Besides technology, there is a need for alternative approaches to promoting physical activity that do not depend on organized sports. These could include community-driven programs, home-based exercise routines, and adaptable outdoor activities. Diversifying physical activity options can help create more resilient systems that can withstand future disruptions.

The study highlights the need for comprehensive public health strategies that prioritize youth physical activity. These strategies should go beyond promoting organized sports and include a wide range of activities that can be easily integrated into daily life. Public health campaigns should emphasize the importance of regular physical activity for both physical and mental health, offering practical guidance on staying active under challenging circumstances.

### 4.3. Psychological Effects of the Lockdown

Physical activity is vital not only for physical health but also for mental well-being. Regular exercise has been proven to alleviate symptoms of anxiety and depression, enhance mood, and improve cognitive function [34]. Sports, in particular, offer additional benefits such as a sense of purpose, structure, and social connection, which are crucial for mental health.

The COVID-19 pandemic disrupted these protective factors, leading to a rise in mental health issues among youth. The loss of daily routines increased social isolation, and uncertainty about the future significantly heightened stress, anxiety, and depression among children and adolescents [35, 36]. According to a study by the Centers for Disease Control and Prevention [37], there was a 24% and 31% increase in emergency department visits for mental health issues among children aged 5–11 and 12–17, respectively, during the pandemic compared to the previous year.

The reduction in physical activity during the pandemic likely worsened these mental health issues. Exercise plays a key role in reducing stress hormones, releasing endorphins, and improving sleep quality [38]. The decrease in physical activity levels during lockdowns deprived many young people of these benefits, which may have contributed to the rise in anxiety, depression, and other mental health problems [39].

Young athletes experienced particularly severe effects. For them, sports are not only about physical activity but also serve as a source of identity, social connection, and emotional support. The cancelation of sports seasons, loss of competition opportunities, and disruption of training routines led many young athletes to feel isolated, anxious, and depressed. The disruption also affected their goals and aspirations, creating a sense of loss and uncertainty about their future in sports.

Addressing these mental health challenges requires a comprehensive approach that promotes physical activity and supports mental health, especially during crises. Public health strategies should focus on encouraging a wide range of physical activities and providing mental health support to help young people navigate disruptions and maintain their overall well-being.

### 4.4. Socioeconomic and Demographic Disparities

The impact of the COVID-19 pandemic on youth physical activity and sports participation was not uniform across all populations. The lockdown exacerbated social and economic disparities, affecting different segments of the youth population in various ways. Socioeconomic and demographic disparities played a significant role in shaping the extent to which different groups of young people were affected. Children from low-income families, minority groups, and rural areas faced greater challenges in maintaining physical activity during the lockdowns compared to their more affluent peers.

One of the key factors contributing to these disparities was access to resources and opportunities for physical activity. Children from higher income families were more likely to have access to private outdoor spaces, such as gardens or yards, where they could engage in physical activity during the lockdown. They were also more likely to have access to digital resources, such as online fitness classes and virtual sports training programs, which could help them stay active at home [35]. In contrast, children from low-income families were more likely to live in small apartments without access to outdoor spaces, making it difficult for them to engage in physical activity. They were also less likely to have access to digital resources or the Internet, further limiting their opportunities to stay active during the lockdown.

Geographic disparities also played a role. Children living in rural areas faced additional barriers to physical activity during the pandemic. In many rural areas, access to sports facilities and recreational spaces was already limited before the pandemic. The closure of these limited facilities during the lockdown exacerbated the problem, leaving children in rural areas with few options for physical activity [40]. Additionally, rural areas often have lower levels of Internet connectivity, making it difficult for children in these areas to access online resources for physical activity.

The pandemic also highlighted racial and ethnic disparities in youth physical activity. Studies have shown that children from minority groups, particularly Black and Hispanic children, were less likely to meet the recommended levels of physical activity during the pandemic compared to their White peers [25]. These disparities are likely due to a combination of factors, including socioeconomic status, access to resources, and cultural differences in physical activity behaviors.

The disparities in physical activity levels and sports participation during the pandemic have important implications for public health and equity. Children from disadvantaged backgrounds are already at a higher risk of developing chronic health conditions, such as obesity and diabetes, due to factors such as poor nutrition and limited access to healthcare. The decline in physical activity during the pandemic may exacerbate these health disparities, leading to worse health outcomes for these populations in the long term [41, 42].

### 4.5. The Crucial Role of Schools and Public Policies

Schools play a central role in promoting physical activity among young people. However, during the pandemic, many schools had to suspend physical education classes, further reducing exercise opportunities for children and adolescents. This reduction not only had an immediate impact on the physical health of young people but could also have long-term consequences, compromising motor development and active lifestyle habits. The closure of schools, which often provide essential opportunities for physical activity, further worsened this situation, creating a growing gap between those who could maintain an active lifestyle and those who were forced into inactivity [26, 43].

To address these challenges, it is essential that public policies implement strategies to reintegrate physical activity into schools, even in emergency contexts. The adoption of flexible physical education programs that can be adapted to different circumstances is crucial to ensure that young people continue to benefit from exercise, regardless of external restrictions [15, 23, 44].

### 4.6. Long-Term Consequences

The COVID-19 pandemic has highlighted the importance of physical activity and sports participation for the health and well-being of youth. The decline in physical activity levels during the pandemic has raised concerns about the long-term health consequences for this generation of young people [45–47]. Physical inactivity during childhood and adolescence is associated with an increased risk of developing chronic health conditions, such as obesity, diabetes, and cardiovascular disease, later in life [48, 49]. The disruption of physical activity during the pandemic may therefore have long-term implications for the health of this generation.

Moreover, the disparities in physical activity levels and sports participation during the pandemic have raised concerns about equity in health. Children from disadvantaged backgrounds were already at a higher risk of poor health outcomes before the pandemic. The decline in physical activity during the pandemic may exacerbate these health disparities, leading to worse health outcomes for these populations in the long term.

To mitigate these long-term consequences, it is crucial to prioritize the promotion of physical activity among youth as the world recovers from the pandemic. This will require concerted efforts from governments, public health authorities, schools, sports organizations, and communities to create supportive environments that encourage active lifestyles. Some potential strategies include

1. Promoting access to outdoor spaces: Governments and local authorities should prioritize the provision of safe and accessible outdoor spaces for physical activity. This includes investing in parks, playgrounds, and sports facilities, particularly in underserved communities.
2. Supporting schools in promoting physical activity: Schools play a crucial role in promoting physical activity among youth. As schools reopen, it will be important to prioritize physical education and extracurricular sports programs. Schools should also be supported in integrating physical activity into the school day, such as through active breaks and active transportation programs.
3. Encouraging digital and home-based physical activity: The pandemic has shown the potential of digital platforms to promote physical activity. Governments and organizations should invest in the development and promotion of digital resources, such as online fitness classes and virtual sports programs, that can be accessed by children and families at home. These resources should be made accessible to all children, including those from low-income families and rural areas.
4. Addressing mental health needs: The mental health impact of the pandemic on youth should not be overlooked. It will be important to provide mental health support to children and adolescents as they return to normal activities. This may include providing access to counseling services, promoting mental health literacy, and encouraging physical activity as a means of supporting mental well-being.
5. Monitoring and research: Ongoing monitoring and research are needed to understand the long-term impact of the pandemic on youth physical activity and health. This will help inform the development of targeted interventions and policies to promote physical activity and mitigate the health consequences of the pandemic.

### 4.7. Limitations

This study has several limitations that should be considered when interpreting the results.

First, the sample size of 211 participants, while sufficient to observe general trends, may not capture the full variability in youth physical activity patterns across different regions and socioeconomic groups. The study was also limited to Northern Italy, which may not be representative of the experiences of youth in other parts of the country or in different cultural contexts.

Second, the study relied on self-reported data, which can be subject to recall bias and social desirability bias. Participants may have overestimated or underestimated their levels of physical activity, particularly in a context where there is social pressure to maintain certain levels of fitness. Additionally, the study did not account for potential external factors that could have influenced physical activity, such as weather conditions, availability of space for home exercise, or family support for physical activity.

The study focused primarily on organized sports and did not fully explore other forms of physical activity that may have been more accessible during lockdowns, such as walking, cycling, or home-based exercises. This could lead to an underestimation of the total physical activity levels among youth during the study period.

Finally, the study did not account for the psychological and social dimensions of physical activity. The pandemic and the associated lockdowns likely had a significant impact on the mental health and social development of young people, aspects that are closely linked to physical activity but were not explored in this study.

### 4.8. Future Directions

Future research should aim to address these limitations by including larger, more diverse samples that can provide a more comprehensive view of youth physical activity during and after COVID-19 lockdowns. To better understand the long-term impact of the COVID-19 pandemic on youth physical activity and subsequent health outcomes across the lifespan, future research should consider several avenues. Longitudinal studies that track physical activity patterns from childhood through adulthood could provide valuable insights into how disruptions like the pandemic influence lifelong health trajectories. Such studies could also examine the persistence of reduced physical activity levels and their association with chronic disease incidence later in life.

Research should also explore alternative approaches to promoting physical activity that are less reliant on organized sports and more resilient to disruptions. There is a need for research that explores the role of alternative physical activities outside of organized sports, especially those that can be performed independently or in small groups. This could include studying the effectiveness of home-based or community-driven physical activity programs that can be maintained during public health crises. Additionally, there is a need to investigate how technology, such as fitness apps and virtual exercise programs, can be leveraged to support physical activity during periods of restricted movement. Investigating the effectiveness of different interventions to promote physical activity during periods of restricted movement could provide insights into more resilient strategies for maintaining youth physical activity in future public health crises.

Furthermore, future studies should consider the broader psychological and social impacts of reduced physical activity during critical developmental periods, particularly how disruptions to sports participation affect mental health and social development. Understanding how physical inactivity during youth affects mental health and social integration, particularly in the context of a global crisis, will be crucial for developing comprehensive strategies to support young people's overall well-being during and after such events.

By focusing on these areas, future research can provide a deeper understanding of the enduring effects of the COVID-19 pandemic on youth physical activity and offer solutions to mitigate these impacts, ensuring that young people can maintain healthy physical activity habits that benefit them throughout their lives.

## 5. Conclusion

This study, conducted across three phases of the COVID-19 lockdown in Italy, demonstrated that youth physical activity was severely disrupted by the suspension of organized sports. Although participation recovered significantly once restrictions were lifted, activity levels remained below the World Health Organization recommendation of 60 min of daily moderate-to-vigorous exercise, and the observed changes were not clinically meaningful. The reliance on organized sports as the predominant form of engagement limited the adoption of alternative modalities such as home-based, individual, or digital exercise, in contrast to reports from other countries. This dependence underscores the structural fragility of a system highly vulnerable to disruptions.

Public health strategies should therefore go beyond reinstating traditional sports programs and promote flexible, sustainable, and resilient models of physical activity that can be maintained during periods of restriction. Integrating accessible home-based activities, leveraging digital platforms, and ensuring equitable access to safe outdoor environments may help safeguard youth's physical activity during future crises. Further research is warranted to investigate the sociocultural and structural factors influencing activity choices and to identify interventions capable of bridging the persistent gap with international health recommendations.

## Data Availability

The datasets generated and/or analyzed during the current study are available from the corresponding author upon reasonable request.
